# Pathological features of tissues and cell populations during cancer cachexia

**DOI:** 10.1186/s13619-022-00108-9

**Published:** 2022-04-20

**Authors:** Daniela Di Girolamo, Shahragim Tajbakhsh

**Affiliations:** 1 Stem Cells & Development, Department of Developmental & Stem Cell Biology, Institut Pasteur, 75015 Paris, France; 2 UMR 3738, CNRS, Institut Pasteur, 75015 Paris, France

**Keywords:** Cancer cachexia, Stem cells, Tissue wasting

## Abstract

Cancers remain among the most devastating diseases in the human population in spite of considerable advances in limiting their impact on lifespan and healthspan. The multifactorial nature of cancers, as well as the number of tissues and organs that are affected, have exposed a considerable diversity in mechanistic features that are reflected in the wide array of therapeutic strategies that have been adopted. Cachexia is manifested in a number of diseases ranging from cancers to diabetes and ageing. In the context of cancers, a majority of patients experience cachexia and succumb to death due to the indirect effects of tumorigenesis that drain the energy reserves of different organs. Considerable information is available on the pathophysiological features of cancer cachexia, however limited knowledge has been acquired on the resident stem cell populations, and their function in the context of these diseases. Here we review current knowledge on cancer cachexia and focus on how tissues and their resident stem and progenitor cell populations are individually affected.

## Background

Cachexia is a condition that results in a significant loss in body mass, muscle wasting, and loss of appetite. It is a frequent side effect of many diseases, including renal failure, various chronic diseases, diabetes, sepsis, ageing, and most commonly, advanced stages of cancer (Fearon et al., 2013; Fearon et al., 2012). Cachexia is considered to be a multi-organ syndrome that affects brain, heart, gut, pancreas, liver, and bone, but most importantly it results from loss of adipose tissue and skeletal muscle (Siddiqui et al., 2020). Specifically, in the context of cancer where up to 70% of patients suffer from cachexia, the loss of skeletal muscle contributes to decreased tolerance of patients to treatments, reduction in the response to therapy, reduced quality of life associated with physical disabilities, and reduced incidence of survival.

Significantly, over 30% of cancer deaths are due to cachexia rather than the tumor itself (Siddiqui et al., 2020). This syndrome usually occurs in stages that are defined by differences in food intake, weight loss, and ability to function. The cachectic condition is complex and can evolve through a spectrum defined as pre-cachexia, cachexia and refractory cachexia, with weight loss ranging from < 5% to 25% (Fearon et al., 2011). In severe stages when muscle wasting is obvious, the condition may be resistant to conventional treatments such as dietary supplementation and nutritional supplements, however, early metabolic changes in pre-cachexia such us impaired glucose tolerance, and clinical indicators (ex. loss of appetite), are already manifested (Fearon et al., 2011). The incidence and severity of cancer cachexia can vary according to tumor type, site and mass, and it is commonly associated with gastric or pancreatic cancer (54–67%), while it is less frequently seen in patients with breast cancer or sarcomas (12–18%) (Cole et al., 2018). Both primary and metastatic tumors can promote cachexia, however surgical removal of the tumor can alleviate the symptoms, while in the case of metastatic cancer, chronic symptoms persist (Biswas & Acharyya, 2020). In spite of numerous clinical trials, to date there are no efficient therapeutic strategies. Additionally, chemotherapy and/or radiotherapy enhance the cachectic syndrome (Aversa et al., 2017; Imai et al., 2019).

The cachectic syndrome is mainly characterised by metabolic dysregulation with an increase in protein degradation and a decrease in protein synthesis. Collectively, these events provoke a negative energy balance due to tumor-secreted molecules and tumor-host interactions (Rohm et al., 2019). In this review, we provide an overview of some of the underlying molecular mechanism and pathophysiology of cancer cachexia in several target tissues and organs and their potential impact on the respective stem cells populations.

## Mechanisms of cancer cachexia: from metabolic dysfunction to inflammation in several target tissues

**Fig. 1:**
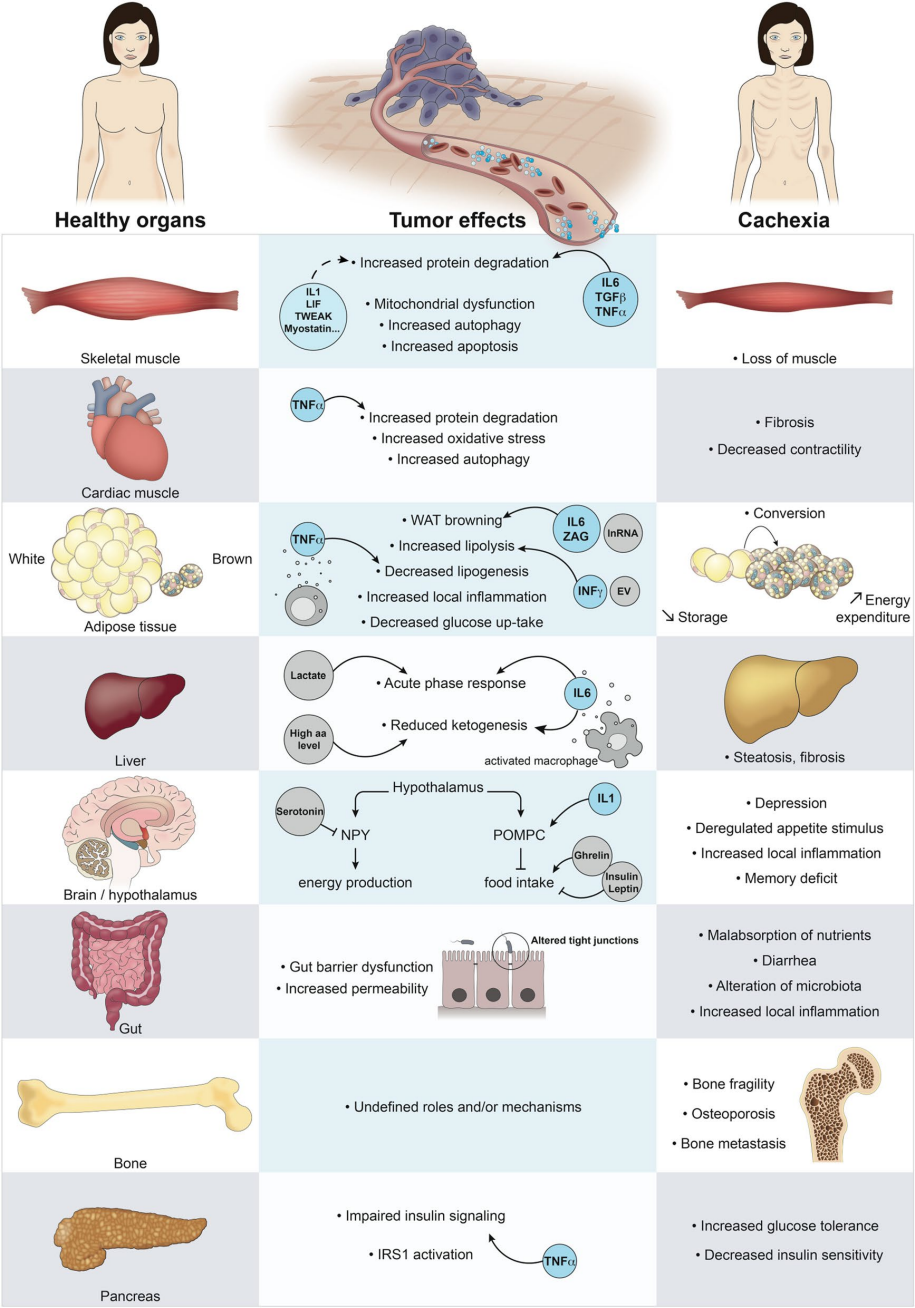
Scheme of the most common target tissues compromised during cancer cachexia. Tumor-secreted factors (pro-inflammatory cytokines and pro-cachectic molecules) contribute mainly to loss of skeletal muscle, cardiac muscle, and adipose tissue. Abnormalities are also found in liver, brain, gut, bone and pancreas. aa, amino acids; IL1, interleukin-1; IL6, interleukin-6; TNF-α, tumour necrosis factor alpha; TGFβ, transforming growth factor-β; LIF, leukemia inhibitory factor; TWEAK, TNF-like weak inducer of apoptosis; IFNγ, interferon gamma; EVs, Extracellular Vesicles; lnRNA, long non-coding RNA; NPY, Neuropeptide Y; POMPC, Pro-opiomelanocortin; ZAG, Zinc-α2-glycoprotein; IRS1, Insulin Receptor Substrate 1

In response to the high metabolic demands of cancer cells, a hyperactivation of the ubiquitin–proteasome and autophagy pathways takes place resulting in compromised tissue integrity and function, particularly in skeletal muscle (Siddiqui et al., 2020). A major driver of the cancer cachexia phenotype is inflammation, where pro-inflammatory cytokines produced by cells of the immune system, as well as by the tumor cells, are released into the circulation and are responsible for the wasting phenotype associated with this condition (Peixoto da Silva et al., 2020). One of the first systemic inflammatory molecules identified in cancer patients is tumor necrosis factor alpha (TNF-α), initially termed “cachectin” (Patel & Patel, 2017). Further studies demonstrated that other inflammatory molecules such us interleukin-1(IL-1) (Laird et al., 2021), interleukin-6 (IL-6) (Narsale & Carson, 2014), interleukin-8 (IL-8) (D. Zhang et al., 2009) and interferon gamma (IFNγ) help to drive the wasting phenotype (Fig. 1) (Matthys et al., 1991).

### Skeletal muscle

**Fig. 2:**
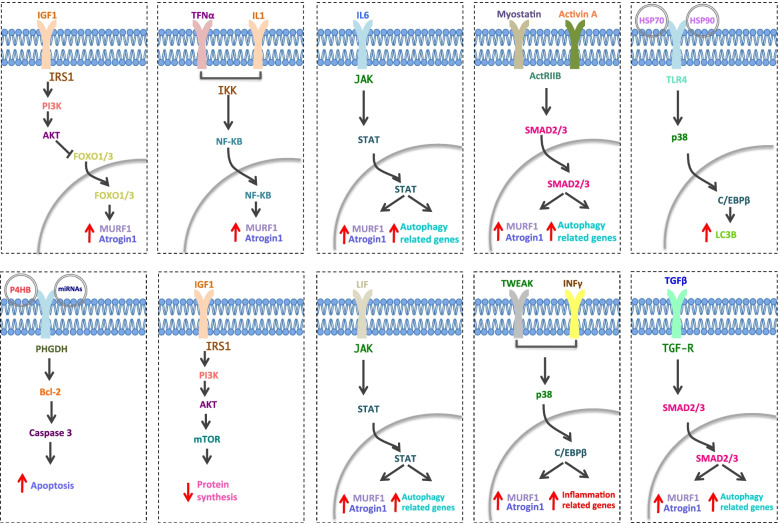
Scheme of the most common signaling pathways active in muscle during cancer cachexia. Several signaling pathways are activated by pro-inflammatory cytokines and tumuor-derived molecules. Protein degradation, through ubiquitin-proteosome pathways, can be activated by Insulin-like growth factor 1 (IGF1)/AKT signaling, Tumor Necrosis alpha (TNFα)/ nuclear factor-KB (NF-KB) signaling, Interleukin1 (IL-1)/NF-KB signaling, Interleukin 6 (IL6)/ Janus kinase (JAK)- signal transducer and activator of transcription proteins (STAT) signaling. Other pathways involved in the activation of the ubiquitin-proteosome pathway are p38/ CCAAT Enhancer Binding Protein Beta (C/EBPβ) and SMAD2/3 signaling induced by TNF-like weak inducer of apoptosis (TWEAK), Interferon gamma (INFγ) and transforming growth factor beta (TGFβ). These pathways all converge to activate the muscle specific ubiquitin ligase Atrogin1 and MURF1 (muscle atrophy F-box protein (MAFBX) and muscle RING finger-containing protein 1 (MURF1)) that ubiquitinate myofibrillar protein, inducing their degradation. The autophagy pathway can also be activated by STAT, SMAD2/3 p38 signaling pathways. Insulin-like growth factor 1 (IGF1), which normally stimulates protein synthesis via AKT and mTOR, is decreased during cachexia. These pathways are generally active in skeletal muscle. The activation of the TNFα/ NF-KB dependent ubiquitin–proteasome pathway is also active in cardiac muscle. Also, the autophagy pathway is activated in cardiac muscle

The extent of skeletal muscle atrophy in cancer can vary considerably depending on the type of tumor, genetic predisposition, reduced food intake, degree of metabolic changes, and type of therapy. A compounding effect ensues as skeletal muscle loss and function can limit the patient's response to therapies (Coss et al., 2018). Generally during cachexia of skeletal muscle, there is an up-regulation of muscle-specific E3 ubiquitin ligases (muscle atrophy F-box protein, MAFbx/atrogin1) and muscle RING finger-containing protein 1 (MuRF1) which ubiquitinate myofibrillar protein for degradation (Glass et al., 2010), thus leading to muscle atrophy. Both atrogin1/MAFbx and MuRF1 are upregulated by FoxO1/3 transcription factors, which in turn are inversely regulated by PI3K-Akt signaling in response to insulin-like growth factor-1 (IGF-1) activity (Sin et al., 2019). Additionally, low levels of circulating IGF-1 and relative insulin resistance have been noted in both humans and mice in cachectic conditions (Fig. 2) (Asp et al., 2010). However, recent studies have demonstrated that biopsies from cachectic patients affected by pancreatic ductal adenocarcinoma (PDA) do not show any significant up-regulation of Atrogin1 and MuRF1 compared to C26 or LLC1 tumor-bearing mouse models that have been used extensively (Talbert et al. 2019). A new mouse model of PDA that recapitulates more faithfully the cachectic signature found in cancer patients was reported by these authors (Talbert et al. 2019). These findings provide exciting new possibilities to re-evaluate the molecular mechanisms responsible for the cachectic phenotype in muscle and others tissue.

Another transcription factor involved in upregulation of the ubiquitin proteasome pathway is NF-κB, which stimulates MuRF1 expression after tumor induction in mice following the injection of Lewis lung carcinoma (LLC) cells (Fig. 2) (Cai et al., 2004). Several studies also demonstrated that in cancer patients experiencing cachexia, autophagy is often up-regulated. Notably, cachexia can be mediated by autophagy in lung cancer patients where BCL2 adenovirus E1B 19-kDa-interacting protein 3 (BNIP3) and Microtubule Associated Protein 1 Light Chain 3 Beta (LC3B) are the major players (Tardif et al., 2013). Accordingly, Beclin-1 and LC3B II proteins were reported to be increased in skeletal muscles of cancer patients, however p62 (classical receptor of autophagy) protein levels were increased in cachectic muscle suggesting that the decreased lysosomal capacity might reflect increased unprocessed autophagosomes in these patients (Aversa et al., 2016). Therefore, some patients accumulate autophagosomes in muscle suggesting that in these patients the autophagy process might be incomplete (Penna et al., 2013; Aversa et al., 2016; Pigna et al., 2016). Contradictory observations have been reported regarding the protein synthesis pathways in cachexia. Although it is generally accepted that there is impaired anabolic capacity during cancer cachexia, some studies demonstrated that in some tumors, protein synthesis is not altered, or is even enhanced, compared to control (Penna et al., 2019). Possible explanations for the discrepancies found in the protein synthesis pathways might arise from the use of different animal models employed (mouse *vs* rat) or different cell lines used to induce cachexia (LLC1, C26, AH-130 hepatoma). Additionally, the severity of cachexia might impact on the differences between different studies.

On the other hand, considerable information is available on the role of pro-inflammatory cytokines in skeletal muscle. In particular, TNFα, transforming growth factor-β (TGFβ) and IL-6 promote muscle wasting. Other cytokines including IL-1, leukemia inhibitory factor (LIF), and TNF-like weak inducer of apoptosis (TWEAK), as well as members of the TGFβ superfamily, including Myostatin and Activin A, were shown to induce muscle catabolism through the activation of NF-kB, p38 mitogen-activated protein kinase (MAPK) and JAK/STAT pathways (Sin et al., 2019). In particular, myostatin and activin A, have also been shown to enhance loss of muscle mass through the Myostatin/Activin receptor type IIB (ActRIIB). Myostatin (GDF8, growth/differentiation factor 8), inhibits myoblast differentiation and, in response to inflammatory signals, stimulates FoxO upregulation which is followed by activation of ubiquitin proteasome pathways (Wildi et al., 2001; Trendelenburg et al., 2009). Increase in muscle mass is observed in *Myostatin* depleted mice, whereas overexpression of Myostatin showed profound muscle loss in mice pointing to a role for this molecule in negatively regulating muscle growth (Trendelenburg et al., 2009; Zimmers et al., 2002). Accordingly, blocking ActRIIB improves cachexia in tumor bearing mice through abolition of ubiquitin–proteasome pathway activation and the induction of muscle-specific ubiquitin ligases (Figs. 1 and 2) (Zhou et al., 2010).

Interestingly, inflammatory molecules are also known to induce mitochondrial dysfunction in skeletal muscle through the activation of NF-kB, JAK/STAT and SMAD pathways. Specifically, TNFα can reduce muscle ATP synthesis and decrease mitochondrial activity in LLC1 tumor bearing mice (Carson et al., 2016). Furthermore, it was shown that TNFα activates a futile cycle between fructose 6-phosphate and fructose 1,6-bisphosphate which results in a relative increase in energy expenditure and heat production in the C2C12 muscle cell line (Zentella et al., 1993). Therapeutic strategies that target mitochondria might represent a viable approach to combat cancer cachexia. However, several compounds have been tested, but none directly improved mitochondrial efficiency yet, and only partial amelioration of the phenotype has been reported (Penna et al., 2020).

In other studies, it was reported that Extracellular Vesicles (EVs), which can contain proteins, mRNA and microRNA that can be internalised by recipient cells and influence their biological functions, play an important role during cancer-associated cachexia. In particular, high levels of Hsp70 and Hsp90 associated with EVs have been found to be sufficient to induce muscle wasting in several cachexia-inducing cancer cells, by activating TLR4 in muscle cells (Zhang et al., 2017). Additionally, EVs secreted by esophageal squamous cell carcinoma (ESCC) cells induced apoptosis of muscle cells through release of prolyl 4-hydroxylase subunit beta (P4HB) that activates the ubiquitin-dependent proteolytic pathway. This in turn regulates the stability of phosphoglycerate dehydrogenase (PHGDH) and subsequently the anti-apoptotic protein Bcl2 (Gao et al., 2021). Accordingly, down-regulation of Bcl2 and activation of the apoptotic signaling pathway in skeletal muscle were observed in colon cancer cachexia models as a consequence of increased production of cancer-derived exosome containing miRNAs (miR-195a-5p and miR-125b-1-3p) (Miao et al., 2021). Similarly, microvesicles containing miR-21 can promote muscle cell death through activation of Toll-like receptor 7 in cancer cachexia (He et al., 2014). In addition to containing miRNAs, IL-6 can be delivered by EVs to muscle and adipose tissue thereby inducing muscle atrophy and lipolysis via STAT3 (Fig. 2) (Hu et al., 2019). These findings highlight the complex nature of the cachectic process which can potentially mobilise diverse cellular pathways to promote tissue wasting.

The heterogeneity of skeletal muscle tissue is mainly reflected by the composition of different fibre types, which differ in the expression of myosin heavy chain isoforms (MyHC), metabolism, and response to neural stimuli (Talbot & Maves, 2016). Notably, skeletal muscle fibres are classified as slow-type1 (express MYH7) and fast-type2 fibres (further classified as type 2A, 2X or 2B based on expression of MYH2, MYH1 and MYH4 respectively) (Talbot & Maves, 2016). These observations raise the possibility that muscle masses that have diverse properties might be differentially susceptible to the deleterious effects of cachexia.

Given the different composition in fibre types of skeletal muscles in different anatomical locations, which in turn reflect distinct contractile properties, several studies aimed to define the impact of cachexia in different fibre types. However, discordant data have been reported. Some studies in human and animal models of cachexia reported a shift towards an increase in fast myosins type 2A and 2B and reduction of type 1 myosin (Taskin et al., 2014; Diffee et al., 2002). In contrast, other studies demonstrated that the distribution between fibre type 1 and 2 remained unchanged in cachectic patients as well as in mouse models for the disease (Op den Kamp et al., 2015; Martin & Freyssenet, 2021; White et al., 2011). These reported differences might reflect differences in cancer types examined, the factors produced, or the extent of the cachexia phenotype in the respective experimental systems.

### Cardiac muscle

Like skeletal muscle, cardiac muscle function is largely compromised during cancer cachexia. Although less information is available on the mechanisms underlying cachexia in the heart, studies with cancer patients have shown heart failure and arrhythmia as major causes of death (Kalantar-Zadeh et al., 2013). Here again, the ubiquitin proteasome and autophagy pathways are implicated. Specifically, TNFα-dependent NF-kB activation up-regulates the ubiquitin proteasome pathway in cardiac muscle leading to atrophy. In addition, the TNF-α/NF-κB pathway has been proposed to increase glucose oxidation at the expense of lipids, by inhibition of PPARγ coactivator 1-α (PGC-1α) a key transcription factor involved in the upregulation of oxidative metabolism (Li et al., 2001) (Fig. 1). This increase in oxidative stress also stimulates muscle atrophy. Furthermore, several autophagy markers such as autophagy related protein 5 (Atg5) and Beclin1 are induced in cardiac muscle of cachectic patients (Fig. 2).

In the C26 colon carcinoma tumor-bearing mouse model, a reduction in heart mass was associated with reduced cardiomyocyte cross-sectional area. During cancer-dependent cardiac remodelling, re-expression of fetal genes, such us Myosin heavy chain (MyHC)-β, takes place (Belloum et al., 2017). Inflammatory molecules released by the tumor and its microenvironment (ex. IL6 (Saito et al., 1999)) are implicated in the remodelling of gene expression. Fibrosis was also found in the heart of C26 tumor-bearing mice, and this contributes to the heart deficiency (Belloum et al., 2017). Altered mitochondrial structure and function is a common feature of cardiac muscle undergoing cachexia (Belury, 2010). Furthermore, decreased phosphorylation of mammalian target of rapamycin (mTOR), S6 ribosomal protein and eukaryotic initiation factor 4E binding protein-1 (4E-BP1) occur in the *Apc*^*Min/*+^ mouse tumor model and they are responsible for the suppression of protein synthesis (Manne et al., 2013). Cachectic hearts from *Apc*^*Min/*+^ mice also exhibited increased phosphorylation of 5'-adenosine monophosphate-activated protein kinase (AMPK), which can lead to autophagy and inhibition of mTOR (Manne et al., 2013). As adjuvants, chemo- and/or radiotherapy treatments were shown to accentuate cardiac impairments present in cancer patients (Ewer & Ewer, 2015).

### White and brown adipose tissue

One of the major targets during cachexia is adipose tissue, which is often lost even before skeletal muscle, primarily due to reduced food intake and tumor-secreted inflammatory molecules (Fouladiun et al., 2005). The combined action of pro-inflammatory cytokines and negative energy intake result in the promotion of lipolysis and inhibition of lipogenesis (Fearon et al., 2012). Elevated lipolysis in cancer cachexia was also supported by the presence of high levels of circulating free fatty acids, glycerol and triacyclycerol in cancer patients (Das & Hoefler, 2013). TNFα mediates cancer cachexia in adipose tissue by decreasing the expression of glucose transporter 4 (GLUT4), which in turn inhibits glucose transport and lipogenesis. Additionally, TNFα recruits cells of the immune system (ex. monocytes) thereby increasing local inflammation in adipose tissue (Siddiqui et al., 2020). Enhanced lipolysis in white adipose tissue in also mediated by IFN-γ which induces insulin resistance through reduction of glucose uptake (Honors & Kinzig, 2012). An important phenomenon that occurs during cancer cachexia is “browning”, that is the progressive conversion of white adipose tissue, which stores energy as fat, into brown adipose tissue, which uses stored energy for heat production. The high number of mitochondria, and relative high expression of Uncoupling Protein 1 (UCP-1), promotes thermogenesis by uncoupling the electrochemical gradient from ATP generation in brown adipose tissue. This results in increased energy expenditure in cancer patients (Petruzzelli et al., 2014) and is mainly dependent on the secretion of parathyroid-hormone-related protein (PTHrP). This in turn induces UCP-1 (Kir et al., 2014), but it can also be regulated by pro-inflammatory mediators such IL-6 or ZAG (Han et al., 2018). Therefore, white adipose tissue browning contributes to cancer cachexia by increasing systemic energy expenditure (Fig. 1).

Like skeletal muscle, the lipolysis of subcutaneous adipose tissue can be mediated by exosomes. The short lipolytic amino acid peptide adrenomedullin is contained in exosomes secreted by pancreatic cancer cells, which will specifically target adipocytes inducing their lipolysis (Sagar et al., 2016). Accordingly, long non-coding RNA like circRNA can in turn aggravate tumor cachexia by regulating WAT browning. It has been demonstrated that ciRS-133, a circRNA targeting miR-133 (which normally inhibits PRDM16 expression) will absorb miR-133 with relative up-regulation of PRDM16 (Zhang et al., 2019).

### Liver

In cancer patients, the liver generally increases in size and undergoes metabolic remodeling that contributes to energy depletion. Tumor metabolism is essentially dependent on aerobic glycolysis (regardless of oxygen availability), a phenomenon termed “Warburg effect.” (Heiden et al., 2009) which is considered an inefficient way to generate adenosine 5-triphosphate (ATP). The role of the liver during tumor formation and progression is to reuse the lactate produced from tumor glycolysis for gluconeogenesis (Porporato, 2016; Friesen et al., 2015). Hepatic gluconeogenesis can also be activated by high levels of amino acids that are derived from protein degradation in muscle. Further, IL-6 that is produced by activated macrophages, stimulates the liver to induce an acute phase response which in turn drives muscle protein degradation and release of free amino acids, thereby enhancing energy wasting (Friesen et al., 2015) (Fig. 1). Tumor-induced IL-6 also reduces the hepatic ketogenic potential through suppression of the master regulator of ketogenesis PPAR in pre-cachectic mice leading to high levels of glucocorticoids under caloric deficiency. Specifically, by suppression of ketogenesis, tumor-induced reprogramming of hepatic metabolism blocks the capacity of the host to produce available energy sources that compensate for decreased caloric intake. Therefore, the impaired ketogenic response to reduced caloric intake results in a systemic metabolic stress response that blocks anti-cancer immunotherapy (Flint et al., 2016).

In addition, tumor-dependent hepatic metabolic dysfunction is manifested by reduced hepatic very-low-density-lipoprotein (VLDL) secretion and hypobetalipoproteinemia (Jones et al., 2013). Accordingly, hepatic steatosis, which is mainly dependent on decreased use of hepatic triglyceride stores, is also a typical symptom of cancer patients and this impacts on overall energy homeostasis (Martignoni et al., 2005). Indeed, it has been demonstrated that carnitine palmitoyltransferase (CPT) I and II activity and mRNA levels, as well as serum levels of free carnitine and acetylcarnitine, were drastically reduced in cachectic mice. Interestingly, exogenous administration of L-carnitine ameliorates cancer cachexia in C26 colon carcinoma bearing tumor mice by regulating the expression and activity of CPT (Liu et al., 2011). Furthermore, studies using Lewis lung carcinoma mouse model have shown high levels of fibrosis and collagen deposition in liver, therefore contributing to hepatic wasting metabolism (Rosa-Caldwell et al., 2020). These studies point to an intimate regulation of liver and muscle proteins and the production of metabolic by-products during cancer cachexia.

### Brain

A common feature in cancer patients is decreased appetite. Cancer-induced anorexia is not only a consequence of depressive behavior typical in terminal-stage cancer patients, but also of deregulation of the hormonal network that stimulates appetite. Food intake and body energy expenditure are regulated by the hypothalamus via modulation of neuropeptide Y (NPY)/agouti-related peptide (AgRP), which stimulates energy production, and pro-opiomelanocortin (POMC)/cocaine-amphetamine-regulated transcript (CART) neurons that inhibit food intake (Chance et al., 2007; Laviano et al., 2008; Silva et al., 2014).

Food intake can also be stimulated by hormones like Ghrelin, and inhibited by hormones such as leptin and insulin (Coll et al., 2007). A large number of cachectic patients exhibit “Ghrelin resistance” where Ghrelin levels are generally high, yet they do not manifest an increase in food intake (Garcia et al., 2005). Additionally, hypothalamus functions are impaired during cancer cachexia where tumor-induced cytokines such as TNFα, interferon gamma, IL-1, and IL-6 stimulate anorexigenic, and inhibit orexigenic pathways (Tuca et al., 2013). For example, IL-1 hyper-activates the POMC/CART pathway in cancer cachexia. Finally, cancer anorexia can also be regulated by serotonin activation of the melanocortin system (Wisse et al., 2001). Studies using murine-derived neuropeptide-Y (NPY)-secreting hypothalamic cell lines demonstrated that serotonin reduces the expression of NPY and this is accompanied by a relative decrease in food intake (Fig. 1) (Van Norren et al., 2017; Burfeind et al., 2016; Dwarkasing et al., 2015).

### Gut

Patients undergoing radio- or chemotherapy treatments, especially if combined, often develop gut barrier dysfunctions. The decreased number of tight junctions leads to a more permeable gut epithelium than allows translocation of bacteria or their components into other organs, resulting in high levels of inflammation and enhancement of the cachectic phenotype. In the case of colon cancers, the gut barrier is disrupted with tumor growth. Studies using the *Apc*^*Min/*+^ mouse model of colon cancer cachexia demonstrated the association between tumor-mediated disruption of the gut and endotoxemia-inflammation (Puppa et al., 2011). Additionally, cancer-associated gut barrier dysfunction leads to malabsorption of nutrients, diarrhea, and other complications, thus generating a negative energy balance and enhancing the cachectic phenotype (Fig. 1).

Several studies demonstrated that alterations of gut microbiota due to cancer treatments and malnutrition might also have a role in cancer cachexia (Bindels & Delzenne, 2013). In particular, lipopolysaccharides and peptidoglycans released by gut microbiota stimulate Toll-like receptor and facilitate the activation of NF-κB pathway, leading to muscle wasting (Bindels & Delzenne, 2013). Additionally, restoring the lactobacilli levels, through hexogen administration, counteracted muscle atrophy and decreased systemic inflammation in a mouse model of leukemia and cachexia (Bindels et al., 2012). Similarly, it has been demonstrated that *Klebsiella oxytoca,* which is the main pathobiont responsible for gut barrier alterations as well as modifications in host gut epithelial metabolism, is increased during cancer cachexia (Pötgens et al., 2018).

The gastrointestinal tract is responsible for the production of the hunger hormone Ghrelin, and this is elevated in different types of cancers. However, even high levels of Ghrelin fail to stimulate appetite, suggesting that in this context Ghrelin might function to reduce inflammation through stimulation of IL-10, which in turn reduces the level of pro-inflammatory cytokines such us IL-1β, IL-6, and TNF-α. Additionally, Ghrelin blocks muscle catabolism, inhibiting the NFκB dependent ubiquitin–proteasome pathway (Chen et al., 2015). Of note, using the C26 bearing tumor mice model, proton nuclear magnetic resonance metabolomics combined with 16S rDNA sequencing showed decreased levels of two short-chain fatty acids (acetate and butyrate) and a reduction in aromatic amino acid metabolites. The bacterium that was responsible for the decrease in butyrate is a member of the Ruminococcaceae family (ASV 2) (Pötgens et al., 2021).

### Bone

Bone loss during cachexia remains largely unexplored. However, several cancer patients have shown increased risk of bone loss and osteoporosis especially when subjected to radio- or chemotherapy treatments (Monroy-Cisneros et al., 2016). Bone metabolic dysfunction, as well as bone fragility, are now considered to be *bona fide* features of cachexia (Verschueren et al., 2013) albeit its active role in the cachectic phenotype remains an open question. Indeed, the degree of bone loss in colorectal cancer cachexia depends upon the tumor type, burden, and duration of the disease (Bonetto et al., 2017). Additionally, chemotherapy-induced loss of bone could be extremely heterogeneous (Hain et al., 2019). In mice with bone metastasis, osteoclasts mediate the release of TGF-β from the bone matrix thus affecting the intracellular calcium signaling and skeletal muscle functions (Fig. 1) (Waning et al., 2015).

### Pancreas

One of the typical metabolic alterations present in almost all cancer patients is glucose tolerance which is often associated with decreased insulin sensitivity (Tayek, 1992). This phenomenon, that in C26 tumor bearing mouse models manifests even before the cachexia phenotype is observed, is mainly dependent on TNF-α, which directly impairs insulin signaling and IRS-1 activation (Hotamisligil et al., 1994; Asp et al., 2010). The decrease in insulin sensitivity is due to changes in insulin signaling rather than structural changes in pancreatic islets (El Razi Neto et al., 1996). Several tumors overexpress the insulin receptor, and together with high levels of insulin, this results in sustained tumor growth (Ozkan, 2011). Furthermore, impairment of insulin signaling also promotes liver gluconeogenesis thereby enhancing tissue wasting and tumor progression (Porporato, 2016). The importance of insulin signaling in preventing cancer cachexia was demonstrated by studies showing that mice bearing C26 tumors treated with rosiglitazone (insulin sensitizer), and patients treated with insulin itself, showed a reduction in cachexia markers (Fig. 1) (Porporato, 2016; Asp et al., 2011).

## Stem cell deregulation in cancer cachexia

### Skeletal muscle stem cells

**Fig. 3:**
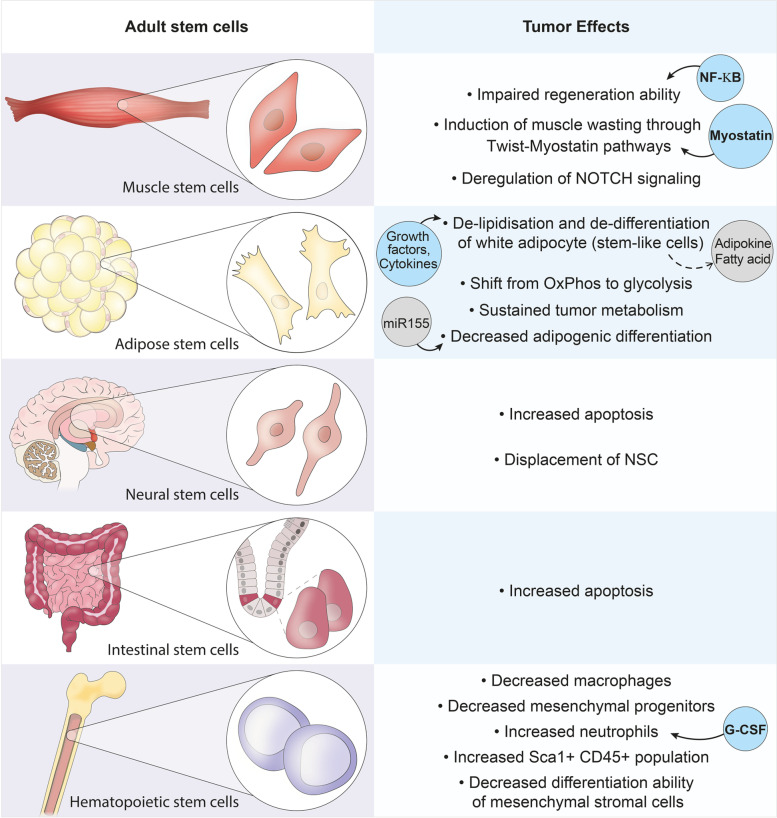
Scheme of some of the most common stem cell populations compromised during cancer cachexia. Release of inflammatory mediators from the tumor alters tissue homeostasis. Resident stem cells appear to modify their behavior when subjected to cachectic conditions. Muscle stem cells appear to have compromised regenerative potential. Adipose stem cells undergo metabolic changes as well as adipocyte shift to a stem-like phenotype. Increased apoptosis in neural stem cells and intestinal stem cells. Hematopoietic stem cells modify their ability to differentiate resulting in the generation of different proportions of immune cells. G-CSF, Granulocyte-colony-stimulating factor; NF-KB, nuclear factor-KB

Myofibre degeneration and regeneration or repair take place after damage to skeletal muscles as they are solicited for labor-intensive tasks. This repair process follows a stereotypical response that involves the mobilisation of muscle stem and niche cells, myoblast amplification and myofibre formation (Evano & Tajbakhsh, 2018). Although muscle stem cells (MuSCs) have been extensively investigated, the relative contribution of the different niche cells to the repair process following muscle damage is less well understood. Cachexia not only impacts muscle fibres, but also muscle stem cells (MuSCs), where aberrant activation of MuSCs leads to an impaired differentiation process during regeneration following injury (He et al., 2013). Some studies have shown alterations of the dystrophin glycoprotein complex and therefore sarcolemma disruption in patients affected by cachexia (Acharyya et al., 2005; He et al., 2013). However, no inflammatory infiltrate has been observed in homeostatic muscle of tumor-bearing animals (Berardi et al., 2008). The recruitment of muscle stem cells in cachectic muscle was reported to be inefficient as they do not reach terminal differentiation due to persistent expression of the paired-homeodomain transcription factor Pax7 that is mediated by NF-κB (Fig. 3) (He et al., 2013). Accordingly, MEK inhibitors prevent the accumulation of MuSCs in C26 tumor bearing-mice and ameliorate the atrophic phenotype of muscle (Penna et al., 2010). Interestingly, the defective differentiation ability is lost when MuSCs are isolated from C26-bearing mice and cultured in vitro, indicating that this phenotype which might implicate metabolic alterations and response to external factors, is reversible (Inaba et al., 2018). Nevertheless, the proliferation and differentiation of MuSCs remained highly compromised in injured C26 tumor bearing mice. This dysfunction correlates with the reduction of macrophages and mesenchymal progenitors, as well as infiltrating neutrophils in cachectic mice following injury of the muscle with the snake venom cardiotoxin (Inaba et al., 2018). In another study, IL4 administration was sufficient to reestablish the number and function of MuSCs in C26-tumor bearing mice. IL4 treatment also improves muscle regeneration in these mice, not only impacting directly MuSCs, but also inducing monocyte differentiation (Costamagna et al., 2020).

As mentioned above, cancer-dependent muscle wasting might be mediated also by MuRF1 and Atrogin1/MAFbx which activate pathways for the degradation of muscle proteins (ex. ubiquitin-proteosome pathway and autophagic-lysosomal degradative pathway). The expression of MuRF1 and Atrogin1 can be induced by Myostatin, which is produced and secreted mainly from skeletal muscle cells and muscle stem cells. The binding of Myostatin with ActRIIB-Alk receptor complex leads to phosphorylation of Smad2 and Smad3, their association with Smad4, and translocation into the nucleus with relative up-regulation of MuRF1 and Atrogin1 (Han et al., 2013; Sartori et al., 2014).

Recent studies demonstrated that the transcription factor Twist1 drives Myostatin synthesis in MuSCs and induces muscle atrophy (Parajuli et al., 2018). Indeed, Twist1 is significantly increased in skeletal muscles of several mouse models of cancer cachexia. Remarkably, conditional deletion of Twist1 in MuSCs is sufficient to suppress cancer-induced muscle wasting (Fig. 3) (Parajuli et al., 2018). Therefore, ActRIIB pathway is implicated in cancer-dependent muscle wasting and its pharmacological block prevents muscle atrophy in several cancer cachexia models (Zhou et al., 2010). Furthermore, blockade of the ActRIIB pathway also results in an increase in the number of quiescent muscle stem cells, and those with strong proliferative capacity (Zhou et al., 2010). Interestingly, CCAAT/enhancer binding protein beta (C/EBPβ), a transcription factor normally expressed in Pax7 + cells and inhibiting myogenic lineage progression, was found to be highly induced in MuSCs in tumor bearing mice. Conditional knockout animals lacking C/EBPβ specifically in Pax7 + cells undergoing cancer cachexia showed increased apoptosis and impaired regeneration. Therefore, stimulation of C/EBPβ expression by IL-1β during cancer cachexia appears to promote MuSC survival (Marchildon et al., 2016).

Skeletal muscle stem cell quiescence is maintained in part through regulation by the Notch pathway (Mourikis & Tajbakhsh, 2014). Additionally, Notch signaling is known to regulate cancer initiation and development (Sethi & Kang, 2011). In the context of cachexia, Notch may play an important role in enhancing muscle atrophy by regulating both tumor and muscle stem cell behavior. Notably, using an orthotopic model of sarcoma-associated cachexia, Notch-activating factors were identified in the exosomes of osteosarcoma cells, which activate Notch signalling in muscle-derived stem cells, leading to impairment of myogenesis (Fig. 3) (Mu et al., 2016).

### Adipose stem cells

Adipose cells have been reported to sustain tumor growth and progression, where mature adipocytes and adipose stem/stromal cells appear to crosstalk with tumor cells (Lengyel et al., 2018). These cells secrete several growth factors and cytokines involved in tissue remodeling and repair, and these molecules are responsible for increased vascularization by neo-vessel formation (da Pinheiro et al., 2012). Several solid tumors have shown a shift from white adipocytes to cells that appear to have stem-like properties (Cao, 2019). During this process, de-lipidation and de-differentiation are activated and free fatty acids and adipokines are released (Cantini et al., 2020). Furthermore, ASCs that are in close proximity to tumor cells showed a metabolic shift from OxPhos to glycolysis (Nieman et al., 2011), suggesting that they might play a role in sustaining the tumor (Fig. 3) (Petruzzelli & Wagner, 2016).

Human adipose stem/stromal cells induced to differentiate in co-cultures with the adrenocortical carcinoma cell line (H295R) showed lower levels of proteins normally associated with functional adipocytes, such as adiponectin, Fatty Acid-Binding Protein 4 (FABP4) and Hormone-Sensitive Lipase (HSL). In addition, these cells were smaller in size and had fewer intracellular lipid droplets compared to those cells cultured without H295R cells. Moreover, these changes were accompanied by an increase in glucose uptake and lactate production suggesting a regulatory cross-talk between adipose and tumor cells (Armignacco et al., 2019).

Adipose mesenchymal stem/stromal cells were reported to be multipotent cells that give rise to several differentiated cell types including adipocytes (Tang & Lane, 2012). The C/EBP family of proteins and peroxisome proliferator-activated receptors (PPARs) appear to be the two most critical classes of transcription factors associated with the adipogenic program (Bougarne et al., 2018). Specifically, C/EBPβ triggers transcription of C/EPBα and PPARγ, which in turn induce the expression of fatty acid binding proteins (FABPs) and fatty acid transport proteins (Bougarne et al., 2018). Furthermore, adipogenic differentiation was reported to be blocked by up-regulation of exosomal miR-155 that is internalised in adipose stem/stromal cells where they target C/EBPβ and inhibit C/EPBα and PPARγ activation thus triggering cancer associated cachexia (Fig. 3) (Liu et al., 2020).

### Neural stem cells

Neural stem cells (NSCs) are located in the adult brain in the subgranular zone in the hippocampal dentate gyrus, the subventricular zone around the lateral ventricles, and the hypothalamus (Andreotti et al., 2019). To date, regions of the brain that are known to be affected by cytokines released from cells of the immune system or by the tumor itself incude the postrema/nucleus of the solitary tract (AP/NTS) region located in the caudal hindbrain (Galic et al., 2012) likely due to the lack of a functional blood–brain-barrier in these regions (Broadwell & Sofroniew, 1993). Although little information is available on the behavior of NSCs during cancer cachexia, patients undergoing chemotherapies of radiotherapies were reported to display memory deficits and depressive symptoms (Dias et al., 2014).

Interestingly, use of therapeutic approaches against the proliferation of cancer cells resulted in a decrease in hippocampal NSC proliferation with a relative increase in apoptosis of these cells, and an overall decrease in the production of new neurons (Monje et al., 2002). Additionally, a sub-population of neural progenitor cells expressing doublecortin (DCX) protein and normally present in the region of the brain where neurons are renewed (sub-ventricular zone) was found outside the brain in the tumor microenvironment (Mauffrey et al., 2019). Moreover, in mouse models of prostate cancer, the reduction of DCX + neural progenitors in the sub-ventricular zone was associated with disruption of the blood–brain barrier and the entry into the circulation of DCX + cells that infiltrate the tumor and generate new adrenergic neurons (Mauffrey et al., 2019). These findings demonstrate how a tumor could communicate with an organ at a distal site to recruit cells that help its growth and progression and impact normal tissue function (Fig. 3).

### Intestinal stem cells

Intestinal stem cells (ISCs) are continuously proliferating multipotent adult stem cells that self-renew and are located in the base of the crypts in the adult intestine. They proliferate and differentiate into specialised cells of the intestinal epithelium and are the cells responsible for the maintenance of the gastrointestinal barrier after damage (Hu & Jasper, 2017). A loss of barrier function in the intestinal epithelium has been reported to be a hallmark of cancer. Consistent with a role for IL-6 in the gut, IL-6 knock-out mice do not develop gut barrier dysfunction after damage (Yang et al., 2003). Furthermore, IL-6 is required, but not sufficient for developing cachexia in Apc^Min/+^ mice (Baltgalvis et al., 2008). Although several studies reported gut barrier dysfunction and dysbiosis in patients affected by cancer cachexia, the role of ISCs in this process remains unclear. However, cancer associated treatments, such us radiotherapy, provoke the apoptosis of ISCs thereby compounding the cachexia phenotype (Yu, 2013).

### Hematopoietic stem cells

The function of immune cells in cachexia remains poorly characterised. Monocytes, monocyte derived macrophages and dendritic cells (DC) are mononuclear cells that play key roles in tissue defense and homeostasis through phagocytosis pathogens and damaged materials (Miller et al., 2019). In response to chemokines produced following insults, naïve monocytes proliferate in the bone marrow and migrate into the damaged site where they generally differentiate to DC or macrophages to phagocytose pathogens or damaged material (Hume, 2006; Capoccia et al., 2009). However, resident monocyte and dendritic cells also play roles in maintaining homeostasis in several tissues. For example, muscle tissue homeostasis not only depends on the balance between protein synthesis and degradation, but also on muscle damage and repair, which is sustained by peripheral and resident immune cells (Arnold et al., 2007).

Only a limited number of studies have reported on the changes to skeletal muscle monocytes or macrophages in cancer-dependent muscle wasting after muscle injury (Inaba et al., 2018; Costamagna et al., 2020). Here, the numbers of both macrophages and mesenchymal progenitors, which are known to be essential for muscle regeneration, were reduced in cancer cachexic mice after injury (Inaba et al., 2018). Additionally, the number of infiltrated neutrophils as well as the expression of critical chemokines for muscle regeneration, were reduced in cancer cachexia mice 24 h after muscle injury. These authors also showed that MuSCs from cachectic mice retain their ability to proliferate and differentiate in vitro. Therefore, cancer cachexia appears to compromise muscle regeneration in part through the regulation of immune cells that are essential for the regeneration process (Inaba et al. 2018). Similarly, IL4 treatment improves muscle regeneration in C26-bearing mice by acting on the function of muscle stem cells and also inducing monocyte differentiation (Costamagna et al., 2020).

In the bone marrow, monocyte and neutrophil fates can be altered from their common precursor under different stimuli. Notably, cancer cells produce granulocyte colony-stimulating factor and can increase circulating neutrophils (Jablonska et al., 2017), whereas their number is reduced by chemotherapy (Shitara et al., 2011). The ratio between circulating neutrophils and lymphocytes is considered to be a strong indicator for survival of cancer patients (Grecian et al., 2018). However, other reports showed decreases in neutrophils with cancer and increases with chemotherapy, suggesting that the numbers of these immune cells might depend on the tumor and chemotherapeutic interventions employed (VanderVeen et al., 2020). Moreover, it has been reported that many tumor-derived cytokines counteract the cytotoxic activity of macrophages and lymphocytes, thereby accounting for the tumor evading the immune response (Elgert et al., 1998).

Additionally, macrophages have been considered to be the main source of TNF-α or IL-1, which are mediators of cachexia (Onesti & Guttridge, 2014). The role of T-cells in cancer-induced skeletal muscle wasting has not been investigated yet, although their role in immune suppression during cancer progression has been studied extensively (Almand et al., 2001). In particular, tumor-infiltrating lymphocytes (TILs) are now used as a prognosis (Morgan et al., 2006)(Deschoolmeester et al., 2010) and several studies showed structural and functional defects in T lymphocytes in the tumor microenvironment (Prins et al., 2001).

Other studies also demonstrated high levels of apoptosis in T-cells present in the tumor, as well as of T-cells in the lymph nodes (Rivoltini et al., 2002; De Lima et al., 2005). Interestingly, CD8 + T-cells appear to induce skeletal muscle wasting during chronic cachexia associated with viral infection (Baazim et al., 2019). Protection from wasting and muscle atrophy by CD4( +)CD44(low) naïve T-cells is associated with protection from lymphopenia (Wang et al., 2008). In contrast, in spite of elevated cytokine levels, the pro-inflammatory cachectic environment is not sufficient to induce a significant recruitment of inflammatory cells to the muscle (Berardi et al., 2008). Indeed, cachectic muscle showed a mild modulation of myeloperoxidase activity, a neutrophil marker, reduced number of macrophages in the endomysium, as well as a reduced number of CD3 + lymphocytes.

Finally, Sca-1 + CD45 + hematopoietic stem cells (HSCs) were reported to be present at higher levels in muscle from cachectic mice suggesting an attempt to maintain muscle homeostasis by recruitment and/or activation of stem cells (Berardi et al., 2008). Moreover, studies using a mouse model of pancreatic ductal adenocarcinoma demonstrated that circulating myeloid cells and primarily neutrophils were present in the brain region important for stimulating appetite, thereby compounding acceleration of the wasting phenotype (Burfeind et al., 2020). This process correlates with the observation that tumor-secreted factors induce expansion of myeloid cells by modification in the hematopoiesis of HSCs (Casbon et al., 2015).

In related studies on invasive breast cancer, reprogramming of early myeloid differentiation in the bone marrow was shown to generate immunosuppressive neutrophils (Casbon et al., 2015). Moreover, mesenchymal stromal cells in bone marrow that are multipotent and can differentiate into different cell types (ex. adipocytes, osteoblasts; (Pittenger et al., 1999)), lose their colony-forming ability and differentiation potential after LLC-induced cancer cachexia due to activation of JAK/STAT and glucocorticoid signaling (Yu et al., 2021).

## Conclusions

Cachexia has a dramatic impact on the quality of life of patients with cancer. Treatments for cancer cachexia symptoms remain challenging given the heterogeneity of tumor types, the complex pathophysiology of the disease, and the multiple organs that are targeted. This is also reflected by the relatively slow advance in therapeutic strategies in spite of considerable investments over the last decades. Indeed, the molecular pathways described so far, which involve inter-organ and intracellular signaling with tumor-secreted factors, have been more challenging to unravel. This review highlights the less-studied role of stem cell populations in different organs, and how they impact on the cachectic phenotype. Interestingly, stem cells appear to modify their behaviour when subjected to cachectic conditions, and often the molecular pathways that act at the tissue level also play a role in deregulating stem cell properties thus perturbing tissue homeostasis. Understanding how stem and stromal cells are remodeled in the niche could provide valuable information for developing new therapeutic approaches that target not only the tissue, but also their resident cell populations, to dampen the cancer phenotypes. The manifestation of disease phenotypes in tissues that are not directly harbouring tumorigenic cells highlights the importance of considering inter-organ communication and systemic effects when developing multi-target drugs to combat the deleterious effects of tumors in cancer patients.

## Data Availability

Not applicable.

## Abbreviations

TNF-α: tumor necrosis factor alpha
IL-1: interleukin-1
IFNγ: interferon gamma
MuRF1: muscle RING finger-containing protein 1
IGF-1: insulin-like growth factor-1
PDA: pancreatic ductal adenocarcinoma
LLC: Lewis lung carcinoma
BNIP3: BCL2 adenovirus E1B 19-kDa-interacting protein 3
LC3B: Microtubule Associated Protein 1 Light Chain 3 Beta
LIF: leukemia inhibitory factor
TWEAK: TNF-like weak inducer of apoptosis
EVs: Extracellular vesicles
ESCC: esophageal squamous cell carcinoma
P4HB: prolyl 4-hydroxylase subunit beta
PHGDH: phosphoglycerate dehydrogenase
MyHC: myosin heavy chain isoforms
PGC-1α: PPARγ coactivator 1α
Atg5: autophagy related protein 5
mTOR: mammalian target of rapamycin
AMPK: 5’-adenosine monophosphate-activated protein kinase
GLUT4: glucose transporter 4
UCP-1: Uncoupling Protein 1
PTHrP: parathyroid-hormonerelated protein
VLDL: very-low-density-lipoprotein
CPT: carnitine palmitoyltransferase
NPY: neuropeptide Y
AgRP: agouti-related peptide
POMC: pro-opiomelanocortin
CART: cocaine-amphetamineregulated transcript
MuSCs: muscle stem cells
C/EBPβ: CCAAT/enhancer binding protein beta
FABP4: Fatty Acid-Binding Protein 4
HSL: Hormone-Sensitive Lipase
NSCs: Neural stem cells
DCX: doublecortin
ISCs: Intestinal stem cells
DC: dendritic cells
HSCs: hematopoietic stem cells
